# Rehabilitation of Flabby Ridges for Denture Fabrication: An Enigma for Dental Professionals

**DOI:** 10.7759/cureus.62345

**Published:** 2024-06-13

**Authors:** Swati Sharma, Sarat Ravi Kiran, Rama Shankar

**Affiliations:** 1 Dentistry, Tata Main Hospital, Jamshedpur, IND; 2 Oral and Maxillofacial Surgery, Tata Main Hospital, Jamshedpur, IND

**Keywords:** functional impression, partial edentulism, epulis fissuratum, combination syndrome, flabby ridge

## Abstract

Edentulism can be treated with a removable or fixed prosthesis to improve aesthetics, comfort, and function. The firmness of the residual ridge is crucial for providing sufficient support for soft tissue. However, a flabby ridge lacks adequate tissue support, which poses challenges for clinicians during impression-making and prosthesis fabrication. Conventional prosthodontic approaches are typically used to manage flabby ridges. This article describes two methods for recording flabby ridges: surgical and unique impression techniques. In the first case, the patient was partially edentulous with a flabby ridge in the lower anterior region. This was removed surgically, and a prosthesis was made conventionally. There has been no relapse in treatment after a follow-up of almost one year. In the second case, the flabby ridge was there in the upper anterior region, which was first excised surgically, but the residual ridge was there; hence, the impression was made by the window technique. After a follow-up of almost nine months, there was a relapse in treatment where a flabby ridge was present in the anterior as well as posterior regions. Hence, again, a window impression was recorded, and a new prosthesis was given to the patient, which yielded satisfactory results. In this article, we have presented two cases of flabby ridges that were successfully treated with surgical and non-surgical techniques followed by special impression techniques. Such analogous cases provide dental professionals with insight into the different lines of treatment, relapse, and further management.

## Introduction

Edentulism is a primary global public health concern due to its high prevalence, which exceeds 10% in adults aged 50 years and older, and the resulting disability. Edentulism is treated with fixed (tooth-supported or implant-supported) and removable prostheses. The goal is to improve aesthetics, comfort, and function by replacing missing dental and alveolar structures with stable prostheses [1]. The success of a complete denture prosthesis is often determined by its retention, stability, and support during use. Ideally, the residual ridge should be covered with a 1.5-2 mm layer of the masticatory mucosa to provide sufficient soft tissue support for the denture. In cases of a flabby ridge, the normal tissue support is absent, and instead, there is highly movable soft tissue on the surface of the alveolar ridge. This condition is known as a flabby, displaceable, or fibrous ridge. It leads to bone loss in the anterior maxilla, with fibrous tissue overgrowth replacing the alveolar bone. Studies have shown this condition affects up to 24% of edentulous maxillae and 5% of edentulous mandibles [1, 2]. 

Flabby tissues present a challenge for clinicians as they are displaceable due to fibrous tissue, making it difficult to take impressions and construct complete dentures. According to MacEntee, complete denture support is compromised if the flabby ridge has more than a 2 mm displacement under pressure. An impression technique is needed to compress non-flabby tissues for optimal support without displacing flabby tissues. Implant treatment can be considered if significant resorption compromises prosthetic retention [3]. The treatment of flabby ridges involves several phases, including tissue conditioning, surgical removal of fibrous tissue, implant-retained prosthesis, special impression techniques for compromised ridges, and removable and conventional prosthodontics without surgical intervention. Surgical removal of the flabby tissue can result in a firm ridge but can increase the bulk of denture material, eliminate stress-absorbing soft tissues, and lead to trauma of the underlying tissues, which can further increase bone resorption. Conventional prosthodontic approaches are more commonly used in managing flabby ridges, including special impression techniques and the balancing of occlusal loads. This article describes two different methods: surgical and impression techniques for recording flabby resorbed ridges [1-4].

## Case presentation

Case one

A 45-year-old woman presented to the dental outpatient department with the chief complaint of difficulty chewing and requesting a dental prosthesis. Upon dental examination, it was discovered that the patient was partially edentulous. Due to chronic periodontitis, the patient had no upper anterior teeth and mobile lower anterior teeth. The lower anterior teeth were unsuitable for a partial denture due to loose teeth and gingival tissue. The treatment plan involved the removal of the loose teeth. Subsequently, the patient was examined after 10 days, and it was observed that the wound had healed well, with significant flabby tissue in the lower anterior ridge. 

**Figure 1:**
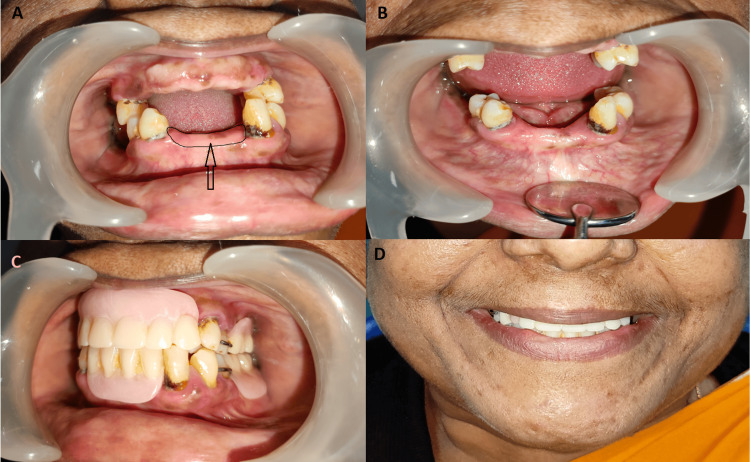
A: flabby ridge in the lower anterior region (marked and shown with a black arrow); B: firm ridge after surgical excision (black arrow); C: partial denture in occlusion; D: the smile of the patient after the insertion of the denture, revealing a good aesthetic outcome.

As the tissue was too displaceable for denture fabrication and retention, surgical excision of the flabby ridge was performed to achieve a better outcome for denture fabrication. The procedure involved giving two elliptical incisions on the ridge in a V shape; subperiosteal dissection was done to expose the underlying bone; and excessive flabby tissue was excised. Bleeding was controlled. The tissues were approximated with the help of 4-0 Vicryl sutures. The patient was recalled for follow-up visits, during which it was noted that the tissues had healed well and exhibited improved firmness. The denture was then fabricated conventionally, and regular follow-up visits showed no relapse or recurrence of flabby tissue even after one year (Figure 1). The patient is currently wearing the partial denture satisfactorily.

Case two

A 55-year-old woman visited the dental outpatient department complaining about her unstable upper denture and requested a replacement. She had been wearing dentures for the past 18 years, with the upper arch completely edentulous and the lower arch partially edentulous; despite initially satisfactory denture wearing, the development of a flabby ridge led to instability and difficulty chewing. The upper anterior region was flabby and highly mobile from the right to the left lateral incisor, making impression-making and subsequent steps unfeasible. Surgical excision was attempted but was not completely successful, leaving residual flabby tissue. 

**Figure 2:**
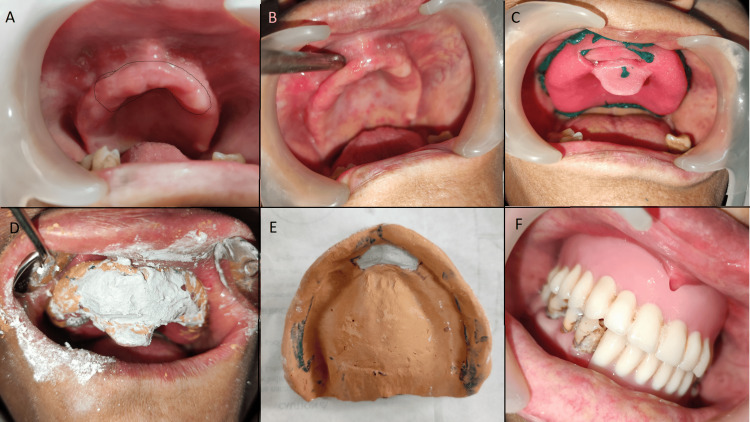
A: flabby ridge in the maxillary anterior region (marked with a black line); B: residual flabby ridge after surgical excision; C: impression making with the window technique; D: paint-on technique in the flabby region in the mucostatic state; E: final two-step impression as a single impression; F: image taken post insertion of the denture intraorally.

A conventional denture fabrication was impossible due to the flabby ridge, so a special impression technique for the flabby ridge area, known as the window impression technique, was used to plan the treatment. The denture was delivered to the patient, and subsequent follow-ups showed satisfaction for up to nine months (Figure 2). 

**Figure 3:**
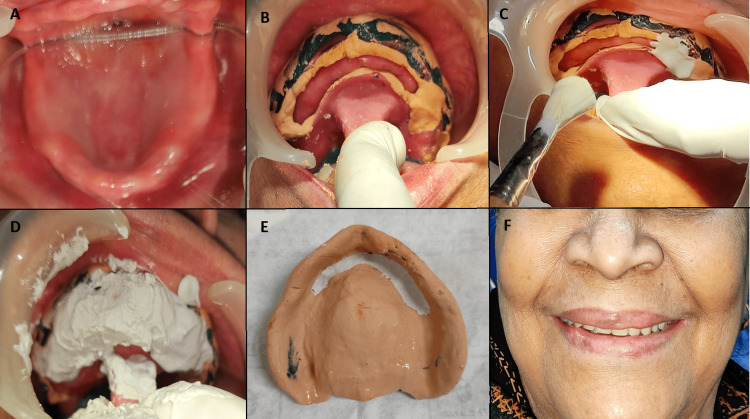
A: flabby ridge in the maxillary anterior region (marked with a black line); B: residual flabby ridge after surgical excision; C: impression making with the window technique; D: paint-on technique in the flabby region in the mucostatic state; E: final two-step impression as a single impression; F: image taken post insertion of the denture intraorally.

However, after nine months, the flabby ridge was recurring in the same anterior region, extending to the posterior region. The relapse most likely occurred because of the residual ridge and occlusal forces, which led to the formation of a flabby ridge. Additionally, the patient mentioned wearing an old prosthesis that was not made with the window technique and put unnecessary pressure on the residual ridge. Although the retention of the upper complete denture was not compromised, the patient was still satisfied. After almost a year, the window impression technique was repeated, including the window's anterior and posterior flabby areas. A new denture with adequate retention and stability was delivered to the patient (Figure 3). 

## Discussion

Flabby ridge is commonly found in the elderly, with no sex predilection. Contributing factors include long-term denture wear without maintenance, trauma from the denture base, anterior occlusal overload, epulis fissuratum, salivary dysfunction, smoking, malocclusion, poor systemic health, unplanned and uncontrolled extractions (uncontrolled medical conditions or locally acute uncontrolled infections), ridge resorption, combination syndrome, prosthetic stomatitis, angular cheilitis, and fibrous hyperplastic lesions [1-4]. Flabby ridges typically occur when an edentulous ridge opposes natural mandibular teeth in the anterior region. These teeth, without proper post-occlusal support, may cause trauma to the maxillary anterior ridge, resulting in overload, severe bone loss, and fibrous hyperplastic tissue formation [5]. E. Kelly coined the term "combination syndrome" to describe a condition characterized by various clinical features. These features include bone loss from the anterior part of the upper jaw ridge, overgrowth of the tuberosities, papillary hyperplasia in the hard palate, protrusion of the lower front teeth, and bone loss under the partial denture bases, which can lead to prosthetic stomatitis [6]. Both presented cases involved females aged 55 and 65 years. In the first case, the etiology of the flabby ridge was malocclusion and unplanned and uncontrolled extractions; in the second case, the etiology was long-term denture wear, trauma from the denture base, and anterior occlusal overload. Histological examination shows fibrosis, inflammation, and resorption of the underlying bone. 

The inspection may be difficult as the color and texture of the tissues are similar to normal unless they are swollen. In a study by Massad et al., they ranked the displacement of flabby tissue after tactile assessment as follows: attached (low mobility, low displacement), average (clinically acceptable), and high mobility, high displacement [7]. They found that hyperplastic tissues moved more than 2 mm with light pressure, which was ranked as high mobility, high displacement, and very difficult to treat in certain cases. The real challenge arises when making new dentures if the flabby ridge is improperly treated. The flabby tissue is compressed during impression-making and will later tend to recoil and dislodge the overlying denture under a masticatory load. This leads to a loss of peripheral sulcular depth that aids in the border seal, causing poor retention, support, and stability of complete dentures [1-5]. An unstable prosthesis will accelerate bone resorption. This is why restoring healthy osteo-mucus support is the preliminary condition for an efficient treatment. Thus, over the years, multiple impression techniques have been proposed to manage a flabby tissue ridge, which will support the flabby tissue but at the same time will not displace it [8]. 

Managing flabby tissues depends on their severity, type, and underlying causes. Treatment usually involves several phases, either separately or at the same time. This can include a conservative approach using mouthwash, nutritional supplements, correcting pressure areas, and addressing occlusal disharmonies through clinical remounting and restoring the vertical dimension [1-5, 8]. The prosthesis should be removed from the mouth for at least eight hours a day for a few days before starting the appropriate treatment. It can also involve relining the old prosthesis with tissue conditioner, which acts as a cushion, absorbs occlusal loads, and enhances their distribution to the tissues. Additionally, soft tissue massage two or three times a day can help recover blood supply and stimulate healing of the inflamed mucosa. The tissue conditioner should be changed every 72 hours [1, 9]. If the condition persists after conservative management, other methods are employed for treatment, such as special impression techniques, conventional prosthodontics without surgical intervention, surgical removal of fibrous tissue before conventional fabrication, and implant-fixed or removable prosthetic treatment [2-5]. Sometimes, augmentation and overdentures may be necessary. Each technique has its advantages and shortcomings. In both presented cases, the flabby ridge was not manageable with a conservative approach or unique impression technique, so a surgical option was chosen [8,9].

Surgical methods for treating soft tissue excess include using a scalpel, injecting a sclerosing agent, and ridge augmentation with hydroxyapatite or a piece of bone from the ribs and hip; if there is a bony deficiency, a graft may be necessary. If there is no bony deficiency, surgical excision may be sufficient. The impression-making procedure can be done three to four weeks after surgery, and prosthodontic treatment can be performed conventionally [10]. The advantage of the surgical technique is that it provides a firm denture-bearing area. Removal is contraindicated in circumstances where little or no alveolar bone remains in patients who are unwilling to undergo surgical treatment. Limitations include the chances of a decrease in vestibular height requiring an additional surgery of vestibuloplasty; the discrepancy will have to be balanced by the base of dentures, implying its thickening, and the simultaneous increase in weight and volume, which eliminates stress-absorbing soft tissues, leading to trauma to the underlying tissues [10]. 

In the first case, surgical removal of the flabby ridge yielded a firm area, and there was no relapse. We then proceeded with making the prosthesis conventionally, which was successful. In the second case, we surgically removed excessive flabby tissue, but residual flabby tissue remained. We used Watson's window technique, where, first, an initial impression was taken using alginate in a perforated stock tray [11]. A special tray with a spacer was used for the cast. The border molding and secondary impression were made using green stick compound and zinc oxide eugenol for non-displaceable tissue. A window was cut in the anterior region over the flabby ridge. The impression was re-inserted in the mouth, and plaster of Paris was painted over the displaceable flabby ridge using the same technique. The final prosthesis fabrication was done conventionally. However, after nine months, there was a recurrence of the flabby ridge in the anterior and posterior regions, possibly due to the use of dentures causing inappropriate forces on the ridge. We repeated the window impression technique, including the anterior and posterior flabby areas in the window, and delivered a new denture with adequate retention and stability to the patient.

In cases where conservative, surgical, and conventional methods are ineffective, implant treatment may be considered a last resort. Implant-supported prostheses provide better retention, stability, and oral function. Reports indicate that the success rates for maxillary implants can be as low as 78.7%, possibly due to the placement of shorter implants into highly vascular, poor-volume, low-density bone [2-5]. Other factors to consider include the patient's general health, the risk of surgical complications or implant failure, patient convenience, discomfort, financial burden, and the prosthodontist's skill. Implant prostheses rely on the underlying bone for support and exert higher bite forces than traditional ones, leading to significant biomechanical stress in the anterior maxilla. Hence, in conventional prosthodontics, with or without surgical intervention, surgical excision followed by conventional methods or special impression techniques may be chosen as the treatment of choice [10]. All impressions for complete dentures can be categorized into three types when considering the impression techniques described in Table 1. 

**Table 1:** Types of impressions to record the denture-bearing surfaces

Mucostatic technique (non-displacive) [12]	Mucocompressive technique (displacive) [13]	Selective pressure impression technique [14]
The denture-bearing area at rest is recorded without displacement. When the denture is closely adapted to the underlying tissues at rest, it is theoretically more retentive. However, this may result in an uneven distribution of occlusal forces across the denture-bearing area.	Compress the loose, flabby tissue to provide functional support by replicating the ridge contour under occlusal forces.	When making dentures, some tissues with dentures are displaced while others are not. To create an accurate impression, a custom tray that fits closely and high-viscosity impression material are used. This compresses the soft tissues at the vibrating line on the palate, while the tightly bound tissue on the hard palate is unaffected.

Currently, the reported studies do not clearly support the excellence of either of these techniques over the other. Prosthodontic literature has documented various impression techniques for overcoming the problem of the flabby ridge described in the subsequent table (Table 2). 

**Table 2:** Various impression techniques documented on the flabby ridge to date

Scientist and year	Technique
Liddledow, 1964 [15]; Crawford et al., 2005 [16]; Magnusson et al., 1986 [17]	Two different impression materials are used in a custom tray: plaster of Paris is used over flabby tissue, and zinc oxide eugenol is used over normal tissue.
Osborne, 1964 [18]	Describe the special impression technique in which two overlying impression trays were used to record maxillary arches with displaceable anterior ridges. This method maintains the contour of the easily displaceable tissue while recording the rest of the denture-bearing area.
Watson et al., 1970 [11]	They explained the "window technique," which involves utilizing a custom tray with a window over the flabby tissues. First, a mucocompressive/mucodisplacive impression is made of the normal tissues using zinc oxide and eugenol. Once set, a low-viscosity mucostatic impression plaster or any light body impression material is painted onto the flabby tissues through the window, applying little or no pressure. After it has been set, the entire impression is removed.
Khan et al., 1981 [19]; Walter, 1973 [20]	The custom tray is modified with posterior handles and an anterior opening for unsupported tissue. A spacer is adapted over the primary cast, except in the region of flabby tissue. This technique is similar to the window technique. Walters' technique is also identical.
Devlin, 1985 [21]	The author describes a precise modification of the Osborne approach by using palatal splinting with a two-part tray system.
Watt and McGregor, 1970 [22]	The impression compound is applied to a modified custom tray and compresses the "normal tissues" while avoiding displacement of the "flabby tissues." A wash impression is made with zinc oxide and eugenol over the manipulated impression compound.
Filler, 1971 [23]	The author has explained a method involving two impression trays. The first tray uses light body impression material for the corrective wash. In the second tray, adhesives are applied to areas not covered by the first impression, and impressions are taken. The two trays are held tightly together, and the final impression is removed as a single unit.
Yazdanie and Hobkirk, 1997 [24]	A single custom tray is used, and the secondary impression is recorded with heavy-bodied addition silicone. Relief holes are made, and the wash impression is recorded using light body impression material.
Massad et al., 2006 [7]	In this technique, a final impression is made in a single visit using a perforated stock tray. For this, spherical pieces of material are placed in one in the anterior region, one in each posterior region, and one in the palatal area. Then, the tray is placed in the patient’s mouth, allowing for 2-3 mm of space. Tissue stops are created with heavy viscosity impression material, which is then allowed to set in the patient’s mouth; border molding is done using heavy and light body impression materials.
Allan, 1997 [25]	The "splint method" is used when tissues are excessively flabby. A loosely fitting tray or a special tray with heavy relief over the flabby area is used to create an impression. The plaster is mixed, applied over the flabby area to a thickness of about 3 mm, and allowed to set. The tray is then filled with a second plaster mix, and the impression is made. The initial plaster coating over the flabby areas acts as a splint and is removed with the second impression.
Lynch and Allen, 2003 [26]	For the wash impression, a modified custom tray was used with an impression compound and zinc oxide/eugenol. The buccal shelf area acted as a stopper, and the remaining borders were recorded using a selective pressure technique and green stick compound. The final impression was recorded using impression silicone.
Applegate, 2009 [27]	Using fluid wax in an impression allows for easy control to achieve maximum coverage and accurately determine the extent of the mucobuccal folds. It can also apply pressure to load-bearing areas, such as the buccal shelves and slopes of residual ridges in the mandible.
Tan et al., 2009 [28]	A functional impression technique using fluid wax is recommended to capture the primary and secondary load-bearing areas without distorting the ridge. First, create a custom tray with a window over the displaceable mandibular ridge. Then, melt impression wax in a water bath and apply it to the borders of the tray until a glossy surface is visible. Apply the adhesive and allow it to dry. Next, place the impression tray on the ridge and inject vinyl polysiloxane impression material over the window. This method creates minimal pressure and accurate details, is resistant to distortion, and is easy to handle.

After the impression, the centric occluding record should be recorded. The tooth arrangement should be in a manner that does not put an undue occlusal load on the ridge and fastens the flabby ridge and bone resorption process. The posterior teeth should be arranged according to the neutral zone, with reduced buccolingual width to decrease pressure on the tissues. The anterior maxillary artificial teeth should be arranged slightly more labially, while the anterior mandibular artificial teeth should be arranged slightly more lingually with no occlusal contact. A lingualized occlusal scheme can also be used for the posterior tooth arrangement, with anatomic teeth for the maxilla and non-anatomic artificial teeth for the mandible [3-5]. The same occlusal schemes were applied in both presented cases for a good prognosis. 

## Conclusions

The presence of flabby tissue presents challenges in rehabilitating completely edentulous patients. Treatment choice depends on the patient's willingness, oral condition, and preference for a fixed or removable prosthesis. Conventional techniques may result in distorted impressions, leading to instability and poor prosthesis retention. Selective pressure or minimally displacive impression techniques can address these limitations. In the Massad class I classification, the window impression technique is applied, and there is no need for an implant-retained prosthesis. The presented cases give dental professionals insight on how to proceed with treatment according to flabby ridge classification and the feasibility and applicability of different methods for a desirable treatment prognosis. It also gives crucial insight for follow-up, outcomes, and managing relapses. It would help clinicians manage analogous occurrences with utmost precision.
